# Editorial: Role of genetics, epigenetics, and environmental factors in human complex diseases from the mitochondrial point of view

**DOI:** 10.3389/fendo.2023.1207351

**Published:** 2023-05-17

**Authors:** Andrea Stoccoro, Adam R. Smith, Fabio Coppedè

**Affiliations:** ^1^ Department of Translational Research and of New Surgical and Medical Technologies, University of Pisa, Pisa, Italy; ^2^ University of Exeter Medical School, Exeter University, Exeter, United Kingdom; ^3^ Interdepartmental Research Center of Biology and Pathology of Aging, University of Pisa, Pisa, Italy

**Keywords:** mitochondria, epigenetics, DNA methylation, mitoepigenetics, environmental factors, oxidative stress

Mitochondria are double membrane organelles involved in several biological pathways, such as ATP generation, apoptosis, and regulation of calcium homeostasis. A peculiar feature of mitochondria is the presence of circular double-stranded DNA molecules inside the organelle, the mitochondrial DNA (mtDNA). The mtDNA includes 37 genes, all required for transcription and translation of electron transport chain subunits in addition to a regulatory non-coding sequence called displacement loop (D-loop) region which regulates mtDNA transcription and replication. Moreover, recent evidence has shown that there are also several non-coding RNAs that are transcribed from the mitochondrial genome (1). Although it is well known that alterations in mtDNA gene expression and replication contribute to the aetiopathogenesis of several human diseases by inducing mitochondrial dysfunction, the mechanisms underlying these events are still not completely understood. Regulation of mtDNA replication and expression is linked to cellular demand, it is strictly dependent on proteins encoded by nuclear genes, to environmental factors as well as to both nuclear and mtDNA genetic variants (2). Moreover, numerous studies are proving that epigenetic mechanisms acting inside mitochondria, including mtDNA methylation and non-coding RNA expression, could also be involved in mtDNA biology, opening a new field of research that has been called “mitoepigenetics” (3). A further layer of mitochondrial epigenetic regulation relies on the numerous mitochondria-derived cofactors necessary for epigenetic mechanisms that regulate both nuclear and mitochondrial gene expression (4).

This Research Topic aimed to collect papers focused on the involvement of mitochondrial dysfunction in human diseases and the role played by genetic, epigenetic, and environmental factors in such dysfunction. We collected five articles focused on the characterization of mtDNA methylation in both physiological and pathological conditions (Devall et al.; Dragoni et al.; Mposhi et al.), on the role of mtDNA in regulating nuclear epigenetic mechanisms (Morin et al.), and in the role of mitochondria-derived oxidative stress in male fertility (Hussain et al.).

Although studies on mitoepigenetic mechanisms are providing new insight into mitochondria biology in both healthy and pathological cellular conditions, the actual presence and function of mtDNA methylation are still heavily debated. This debate is mainly driven by technical challenges in evaluating methylation of the tightly coiled structure of mtDNA that, if not properly addressed, could lead to an overestimation of methylation detection (5, 6). By carefully linearizing mtDNA through DNA sonication, Devall et al. characterized mtDNA methylation by means of targeted bisulfite sequencing in post-mortem brain samples, including superior temporal gyrus and cerebellum of healthy donors. Authors found that the overall methylation level of mtDNA was low but conserved among the subjects and in the different brain areas, with peaks of methylation levels in certain mtDNA regions, including D-loop. The authors also found that mtDNA methylation positively correlated with age and that females had lower mtDNA methylation levels when compared to males. This is the first methylation analysis at the genome-wide level of mtDNA performed in human brain at single base resolution, which provides a map of human brain mtDNA methylation and identifies anthropometric factors that associate with mtDNA methylation levels.

The potential role of altered mtDNA methylation in human diseases has been investigated in two papers collected in the current Research Topic. Dragoni et al. investigated mtDNA methylation in patients affected by Aicardi-Goutières Syndrome (AGS), a rare hereditary encephalopathy caused by mutations in genes that regulate the nucleic acid metabolism. Since the authors previously observed mitochondrial dysfunction in AGS patients-derived cell cultures (7), in the paper published in our collection they aimed to search for alteration in mtDNA copy number and D-loop methylation in peripheral blood of 25 AGS patients and 22 healthy control subjects. They observed that both mtDNA copy number and D-loop methylation levels increased in AGS patients when compared to control subjects. These differences were mainly observed in AGS patients with *RNASEH2B* mutations, the most commonly altered gene in AGS, suggesting that the extent of the mitochondrial deficit in AGS is also dependent on nuclear DNA genetic variants. Mposhi et al. investigated the potential role of mtDNA methylation in metabolic-associated fatty liver disease (MAFLD) pathogenesis, which is characterized by mitochondrial dysfunction and hepatic lipid accumulation. Authors constructed hepatocellular carcinoma (HepG2) cells that stably expressed DNA methyltransferases able to induce mtDNA methylation. In the transgenic HepG2 cells, the mitochondrial gene expression and metabolic activity were impaired and associated with lipid accumulation. To verify if lipid accumulation could induce mtDNA methylation alterations, wild-type HepG2 cells were treated with fatty acids, but no mtDNA methylation alterations were detected. Conversely, mice fed a high-fat diet had increased methylation and expression of the *mt-Nd6* gene when compared to control mice and liver samples of patients with simple steatosis showed increased methylation of the *MT-ND6* gene. Overall, the results of the study by Mposhi et al. suggest that altered mtDNA methylation could associate with mitochondrial dysfunction and lipid accumulation in MAFLD.

A pivotal role in cellular functions, differentiation and response to extracellular environment is played by anterograde, from nucleus to mitochondria, and retrograde, from mitochondria to nucleus, signaling, and dysfunction in these pathways could have pathological consequences (8). In the paper by Morin et al. the cross-talk between the nucleus and the mitochondria has been reviewed from an epigenetic point of view. Particularly, the importance of mtDNA integrity for a proper mitochondrion functioning in producing intermediate molecules for epigenetic mechanisms in the nucleus, such as ATP, α-ketoglutarate, acetyl-CoA and S-adenosylmethionine, has been highlighted. A major focus has been given to the influence of mtDNA heteroplasmy and mtDNA copy number changes on mitochondrially-derived epigenome-modifying molecules in physiological and pathological conditions. Morin et al. reported how mtDNA variations are associated with altered nuclear DNA methylation and post-translational histone tail modifications, likely as a result of the dysregulation of mitochondrial metabolites. The authors also highlighted the environmental factors able to alter mtDNA copy number and heteroplasmy, including alcohol abuse, heavy metals, and lifestyle factors, suggesting that mitochondria may act as environmental biosensors in mediating disease states.

Mitochondria are also the main producers of reactive oxygen species (ROS), which are critical signalling molecules at physiological levels, but are detrimental to cellular components when their generation is compromised (9). The role of oxidative stress derived by impaired mitochondria on male fertility has been reviewed by Hussain et al. In the review, the authors discussed the oxidative stress effects on sperm quality and quantity, highlighting how enhanced ROS production disturbs the cellular constituents, particularly inducing mtDNA damage, and in turn, impairment in fertilization of egg cells. This is of particular interest considering that excessive ROS production can induce altered epigenetic mechanisms during the spermatogenesis process (10) and that are able to impair the mtDNA copy number and global DNA methylation altering semen functional parameters, including sperm motility (11). Finally, Hussain et al. have compiled a list of adverse environmental factors that it would be better to reduce if not avoid in order to decrease the risk of infertility related to oxidative stress.

In summary, the five articles collected in this Research Topic focused on the involvement of mitochondrial dysfunction in physiological and pathological conditions by considering the role played by genetics, epigenetics and environmental factors. These articles could be of interest to researchers involved in the field of mitoepigenetics and in general to scientists working on the role of the complex interaction between nucleus and mitochondria (epi)genetic alterations that underlie mitochondrial dysfunction in human pathologies.

## Author contributions

All authors have made a substantial and intellectual contribution to the manuscript and approved it for publication.
